# Parametric and non-parametric masking of randomness in sequence alignments can be improved and leads to better resolved trees

**DOI:** 10.1186/1742-9994-7-10

**Published:** 2010-03-31

**Authors:** Patrick Kück, Karen Meusemann, Johannes Dambach, Birthe Thormann, Björn M von Reumont, Johann W Wägele, Bernhard Misof

**Affiliations:** 1 Zoologisches Forschungsmuseum A. Koenig, Adenauerallee 160, 53113 Bonn, Germany; 2 Biozentrum Grindel und Zoologisches Museum, Universität Hamburg, Martin-Luther-King Platz 3, 20146 Hamburg, Germany

## Abstract

**Background:**

Methods of alignment masking, which refers to the technique of excluding alignment blocks prior to tree reconstructions, have been successful in improving the signal-to-noise ratio in sequence alignments. However, the lack of formally well defined methods to identify randomness in sequence alignments has prevented a routine application of alignment masking. In this study, we compared the effects on tree reconstructions of the most commonly used profiling method (GBLOCKS) which uses a predefined set of rules in combination with alignment masking, with a new profiling approach (ALISCORE) based on Monte Carlo resampling within a sliding window, using different data sets and alignment methods. While the GBLOCKS approach excludes variable sections above a certain threshold which choice is left arbitrary, the ALISCORE algorithm is free of *a priori *rating of parameter space and therefore more objective.

**Results:**

ALISCORE was successfully extended to amino acids using a proportional model and empirical substitution matrices to score randomness in multiple sequence alignments. A complex bootstrap resampling leads to an even distribution of scores of randomly similar sequences to assess randomness of the observed sequence similarity. Testing performance on real data, both masking methods, GBLOCKS and ALISCORE, helped to improve tree resolution. The sliding window approach was less sensitive to different alignments of identical data sets and performed equally well on all data sets. Concurrently, ALISCORE is capable of dealing with different substitution patterns and heterogeneous base composition. ALISCORE and the most relaxed GBLOCKS gap parameter setting performed best on all data sets. Correspondingly, Neighbor-Net analyses showed the most decrease in conflict.

**Conclusions:**

Alignment masking improves signal-to-noise ratio in multiple sequence alignments prior to phylogenetic reconstruction. Given the robust performance of alignment profiling, alignment masking should routinely be used to improve tree reconstructions. Parametric methods of alignment profiling can be easily extended to more complex likelihood based models of sequence evolution which opens the possibility of further improvements.

## Background

Multiple sequence alignments are an essential prerequisite in alignment based phylogenetic reconstructions, because they establish fundamental homology assessments of primary sequence characters. In consequence, alignment errors can influence the correctness of tree reconstructions [1-3]. To deal with this problem at the level of sequence alignment, different approaches and alignment software tools have been developed, but despite major advances, alignment quality is still mostly dependent on arbitrary user-given parameters, e.g. gap costs, and inherent features of the data [4,5]. In particular when sequences are highly divergent and/or length variable, sequence alignment and the introduction of gaps become a more and more complex enterprise and can currently not be fully governed by formal algorithms. The major problem is that finding the most accurate alignment parameters in progressive and consistency based alignment approaches is difficult due to the incomplete knowledge of the evolutionary history of sequences and/or heterogeneous processes along sequences [6]. As a result, problematic sequence alignments will contain sections of ambiguous indel positions and random similarity.

To improve the signal-to-noise ratio, a selection of unambiguous alignment sections can be used. It has been shown that a selection of unambiguously aligned sections, or alignment masking [7], improves phylogenetic reconstructions in many cases [2,8,9]. However, a formally well defined criterion of selecting unambiguous alignment sections or profiling multiple sequence alignments was not available. To fill this gap, different automated heuristic profiling approaches of protein and nucleotide alignments have been developed. GBLOCKS [10] is currently the most frequently used tool. The implemented method is based on a set of simple predefined rules with respect to the number of contiguous conserved positions, lack of gaps, and extensive conservation of flanking positions, suggesting a final selection of alignment blocks more "suitable" for phylogenetic analysis [10,11]. The approach does not make explicit use of models of sequence evolution and is subsequently referred to as a "non-parametric" approach.

The recently introduced alternative profiling method, ALISCORE [12], identifies randomness in multiple sequence alignments using parametric Monte Carlo resampling within a sliding window and was successfully tested on simulated data. ALISCORE was first developed for nucleotide data, but has been extended here to amino acid sequences. The program is freely available from http://aliscore.zfmk.de. In short, within a sliding window an expected similarity score of randomized sequences is generated using a simple match/mismatch scoring for nucleotide or an empirical scoring matrix for amino acid sequences (see Methods), actual base composition, and an adapted Poisson model of site mutation. The observed similarity score is subsequently compared with the expected range of similarity scores of randomized sequences. Like GBLOCKS it is independent of tree reconstruction methods, but also independent of *a priori *rating of sequence variation within a multiple sequence alignment. Because of its explicit use of, although rather simple, models of sequence evolution, ALISCORE can be called a parametric method of alignment masking.

It has been demonstrated that both methods correctly identify randomness in sequence alignments, although to a very different extent [10-12]. A comparison of their performance on real data is however missing. Both masking methods suggest a set of alignment blocks suitable for tree reconstructions. These alignment blocks should have a better signal-to-noise ratio and this should lead to better resolved trees and increased support values. Therefore, we used these predictions to assess the performance of both masking methods by comparing reconstructed Maximum Likelihood (ML) trees. Additionally, our analyses compared the sensitivity of tree reconstruction given both profiling approaches in relation to different data and alignment methods. Different test data sets were aligned with commonly used alignment software (CLUSTALX 1.81 [13], MAFFT 6.240 [14], MUSCLE 3.52 [15], T-COFFEE 5.56 [16], and PCMA 2.0 [17]). For protein alignments, we used two data sets of mitochondrial protein coding genes that differ in their sequence variability and number of taxa, and an EST data set of mainly ribosomal protein coding genes, including missing data of single taxa. For nucleotide alignments, we tested the performance of ALISCORE and GBLOCKS on highly variable 12S + 16S rRNA sequence alignments (Table 1).

**Table 1:** General overview of used data sets

Data set	Type	No. of genes	Taxon	No. of species	No. of cons. clades	Data source
mtI	AA	11	Eukaryota	17	12	NCBI/SwissProt
mtII	AA	5	Eukaryota	24	15	NCBI/SwissProt
EST	AA	51	Arthropoda	26	7	dbEST; KM/BMvR/FR/TB
12S + 16S	NUC	2	Arthropoda	63	9	NCBI/JD

Data sets used for analyses. mtI: mitochondrial data set I; mt II: mitochondrial data set II; EST: EST data set; 12S + 16S rRNA: mitochondrial ribosomal data set. Type: Kind of sequence type. AA: Amino acid sequences; NUC: Nucleotide sequences. No. of genes: Number of genes per data set. No. of species: Number of species per data set. No. of cons. clades: Number of considered clades (selected). Data source: dbEST: EST database of NCBI; unpublished sequences provided by KM (K. Meusemann), BMvR (B. Reumont), FR (F. Roeding), TB (T. Burmester) and JD (J. Dambach).

## Results

### ALISCORE algorithm for amino acid data

As for nucleotide sequences [12], ALISCORE uses a sliding window approach on pairs of amino acid sequences to generate a profile of random similarity between two sequences. In contrast to the algorithm with nucleotide data, ALISCORE employs the empirical BLOSUM62 matrix, *Q*, (or alternatives of it, PAM250, PAM500, MATCH) to score differences between amino acids, *Q*_*ij*_. Pairs containing indels and any amino acid are defined by using the value of a comparison of stop codons and any amino acid defined within *Q*. The observed score within a window of pairwise comparisons is generated by summing scores of single site comparisons. Starting from a multiple sequence alignment of length *L*, sequence pairs (*i*, *j*) are selected for which the following procedure is executed: In a sliding window of size *w *at position *k*, a similarity score *S*(*k*) is calculated comparing positions (*i*(*k*), *j*(*k*)), ∀ *k *∈ (1, 2, ..., *L*), using the following simple objective function:

Observed scores are compared to a frequency distribution of scores of randomly similar amino acid sequences with length given by the window size. The generation of randomly similar sequences follows the Proportional model [18], which is an adaptation of a simple Poisson model of change probability, adapted for observed amino acid frequencies, but still assuming that the relative frequencies of amino acids are constant across sites:

with *P*_*ij *_(*t*) as the probability of change from amino acid *i *to *j*, *π*_*j *_the frequency of amino acid *j*, *μ *the instantaneous rate of change, and *t *the branch length/time. Different to the algorithm used with nucleotide sequences in which scores are adapted to varying base composition along sequences and among sequences, the frequency distribution of scores of randomly similar sequences is only produced once for amino acid data. The frequency distribution is generated by: 1) collecting frequencies of amino acids of the complete observed data set, 2) generating 100 bootstrap resamples of this amino acid frequency distribution and 100 delete-half bootstrap resamples of each of the 100 complete bootstrap resamples, and 3) by using these 10,000 delete-half bootstrap resamples to generate 1,000,000 scores of randomly similar amino acid sequences with length given by the window size. This complex resampling leads to an even distribution of scores of randomly similar sequences. The frequency distribution of randomly similar sequences is used to define a cutoff *c*(*α *= 0.95) to assess randomness of the observed sequence similarity within the sliding window. Matching indels are defined as *Q*_*ij *_= *c*/*w*. The principle of the complete scoring process is described in [12].

### Testing performance on real data

#### Extent of identified randomly similar blocks

**Figure 1:**
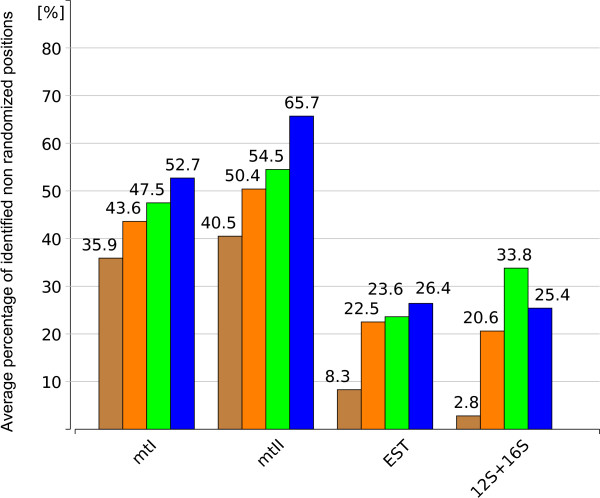
**Percentage of identified non randomized positions**. X-axis: Used data sets. Y-axis: Average percentage of "non randomly similar" positions per data set after alignment masking. GBLOCKS(none): brown; GBLOCKS(half): orange; GBLOCKS(all): green; ALISCORE (blue). A list of all single values is given in the additional file 1.

Compared to GBLOCKS, using ALISCORE resulted in the exclusion of fewer positions in most data sets (Figure 1). GBLOCKS identified fewer randomized positions only for the highly diverse 12S + 16S rRNA data with the GBLOCKS(all) option. For each data set, the percentage of identified randomly similar sections differed on average between 1% and 5% for each multiple sequence alignment when ALISCORE was applied, and between 1% and 9% when GBLOCKS was used. Most alignment sites were discarded by the default option GBLOCKS(none).

#### ML trees and Neighbor-Net analyses

**Figure 2:**
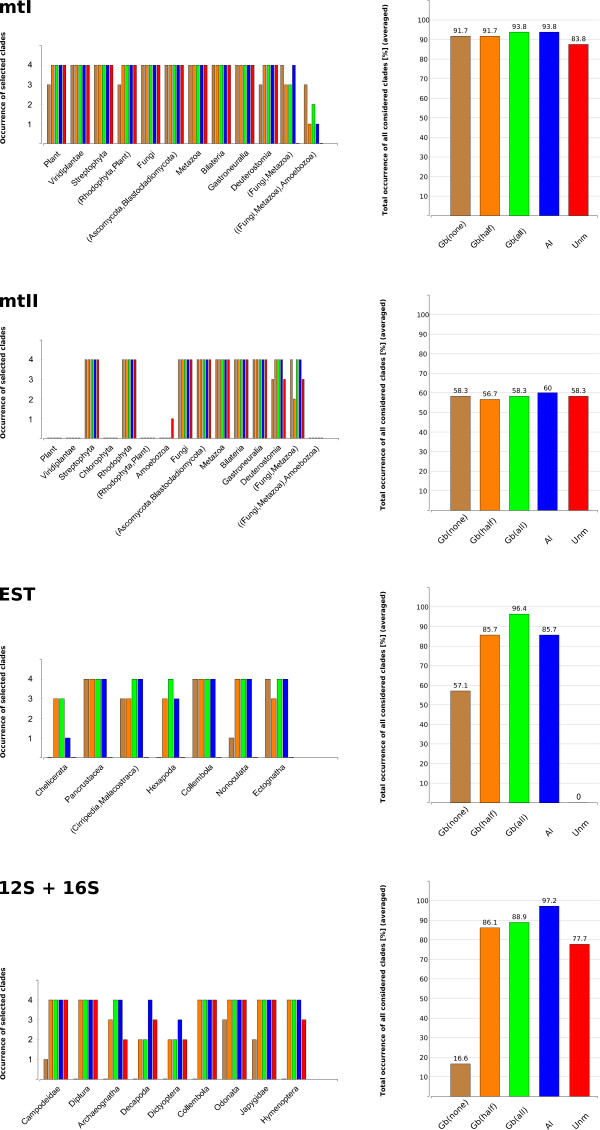
**Average percentage of resolved clades within single data sets**. On the left: Occurrence of selected clades (Table 2-3) of each data set (mtI, mtII, EST, 12S + 16S), inferred from majority rule ML trees. On the right: Total occurrence of all considered clades [%] for each data set, averaged across all four alignment methods. GBLOCKS(none): brown; GBLOCKS(half): orange; GBLOCKS(all): green; ALISCORE: blue; Unmasked: red.

**Figure 3:**
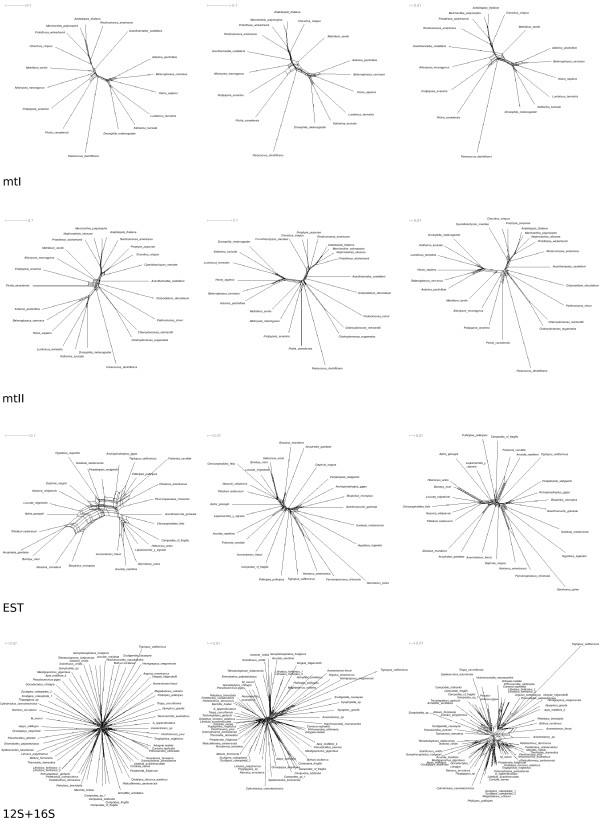
**Neighbor-Net graphs**. Neighbor-Net graphs generated with SplitsTree 4.10 based on concatenated supermatrices of unmasked (left), ALISCORE masked (middle), and GBLOCKS(none) masked (right) data. mtI, mtII and EST networks depend on a T-COFFEE alignment, the 12S + 16S rRNA network on a PCMA alignment. Neighbor-Nets were calculated with uncorrected p-distances. All inferred Neighbor-Net graphs are given in the additional File 1. Tree like structures in these graphs indicate distinct signal-like patterns in the corresponding alignment. Graphs generated from ALISCORE data sets are more tree-like. Lack of information leads to star-like graphs, conflicting signal produces cobwebs.

Resulting ML trees and Neighbor-Net graphs were examined under two different aspects: 1) We compared trees of all unmasked alignments with trees of differently masked alignments per data set to analyze the influence of each masking method on data structure and presence/absence of selected clades (Figure 2). 2) We compared bootstrap values of corresponding trees (Table 2) and Neighbor-Net graphs (Figure 3) of unmasked and differently masked alignments to see if alignment masking improves the signal-to-noise ratio in the predicted way.

**Table 2:** Averaged bootstrap support [%] of selected clades

Data set	Selected clades	Gb(none)	Gb(half)	Gb(all)	Al	Unm
**mtI**						
						
	Plant	63.8	82.0	77.5	81.5	97.0
	Viridiplantae	99.8	99.5	99.8	100.0	100.0
	Streptophyta	100.0	100.0	100.0	100.0	100.0
	(Rhodophyta, Plant)	68.5	92.0	88.5	82.8	96.8
	Fungi	100.0	100.0	100.0	100.0	100.0
	(Ascomycota, Blastocladiomycota)	100.0	100.0	100.0	100.0	100.0
	Metazoa	100.0	99.5	99.3	100.0	85.8
	Bilateria	93.3	100.0	100.0	100.0	100.0
	Gastroneuralia	100.0	100.0	100.0	100.0	100.0
	Deuterostomia	73.0	99.8	100.0	99.8	100.0
	(Fungi, Metazoa)	100.0	62.5	75.0	76.0	0.0
	((Fungi, Metazoa), Amoebozoa)	41.3	17.3	46.3	24.0	0.0
**mtII**						
						
	Plant	0.0	0.0	0.0	0.0	0.0
	Viridiplantae	0.0	0.0	0.0	0.0	0.0
	Streptophyta	100.0	100.0	100.0	100.0	100.0
	Chlorophyta	0.0	0.0	0.0	0.0	0.0
	Rhodophyta	99.5	93.5	98.5	97.5	97.8
	(Rhodophyta, Plant)	0.0	0.0	0.0	0.0	0.0
	Amoebozoa	0.0	0.0	0.0	0.0	14.3
	Fungi	100.0	100.0	100.0	100.0	93.3
	(Ascomycota, Blastocladiomycota)	100.0	100.0	100.0	100.0	96.2
	Metazoa	100.0	100.0	100.0	100.0	100.0
	Bilateria	100.0	100.0	100.0	100.0	100.0
	Gastroneuralia	98.5	100.0	100.0	100.0	88.5
	Deuterostomia	69.3	94.5	98.8	95.5	73.3
	(Fungi, Metazoa)	87.3	70.5	64.8	79.3	67.5
	((Fungi, Metazoa), Amoebozoa)	0.0	0.0	0.0	0.0	0.0
**EST**						
						
	Chelicerata	0.0	50.8	57.5	24.8	0.0
	Pancrustacea	97.8	99.5	100.0	100.0	0.0
	(Cirripedia, Malacostraca)	56.0	58.5	79.5	85.0	0.0
	Hexapoda	0.0	46.5	66.8	52.8	0.0
	Collembola	98.8	99.5	99.8	100.0	0.0
	Nonoculata	18.8	74.5	72.8	84.3	0.0
	Ectognatha	88.0	55.8	76.8	75.3	0.0
**12S + 16S**						
						
	Campodeidae	17.5	89.3	98.8	97.0	99.8
	Diplura	0.0	87.8	96.8	92.5	94.3
	Archaeognatha	0.0	59.8	85.3	75.8	47.5
	Decapoda	0.0	38.5	38.0	81.8	73.3
	Dictyoptera	0.0	38.0	39.3	45.3	49.5
	Collembola	0.0	97.5	99.3	97.5	96.0
	Odonata	57.8	100.0	100.0	99.3	100.0
	Japygidae	29.8	99.5	99.0	100.0	100.0
	Hymenoptera	0.0	88.0	84.3	83.8	24.8

Averaged bootstrap support [%] of selected clades of each data set (mtI, mtII, EST, 12S + 16S) inferred from majority rule ML trees for all GBLOCKS profiles (Gb(none), Gb(half), Gb(all)), ALISCORE (Al), and Unmasked (Unm). Values are averaged across different alignment methods per masking approach.

In general, ALISCORE masked alignments resulted in consistent ML topologies among identical but differently aligned sequence data. The ALISCORE algorithm performed in most cases better or at least equal well than the best GBLOCKS settings (GBLOCKS(all), GBLOCKS(half)). Application of GBLOCKS(none) yielded less congruent trees.

##### Amino acid data

While plants, fungi, metazoans, and included subtaxa were fully resolved in unmasked trees of data set mtI, sister group relationships between major clades (Fungi, Metazoa, Amoebozoa) could not be resolved without alignment masking. If alignments were masked according to the ALISCORE profile, all ML trees showed a sister group relationship between fungi and metazoans. In the case of the T-COFFEE alignment, the Amoebozoa were placed as sister group to Fungi + Metazoa. The alignment masking of GBLOCKS(all) and GBLOCKS(half) led to comparatively resolved topologies. Masking the alignments with the GBLOCKS(none) option reduced signal in the data (Figure 2). Bootstrap values as measurement of data structure increased after alignment masking, in particular for deep nodes (clade (Fungi, Metazoa) and ((Fungi, Metazoa), Amoebozoa), see Table 2). After alignment masking, Neighbor-Net graphs showed less conflict (Figure 3).

For the mtII data set we were not able to recover monophyletic plants and Amoebozoa as sister group to Fungi + Metazoa. The sister group relationship between Fungi and Metazoa was fully resolved in all ALISCORE, GBLOCKS(all), and GBLOCKS(none) masked data sets. GBLOCKS(none) and GBLOCKS(half) masked alignments supported in several instances implausible clades (Figure 2).

Bootstrap support values only marginally increased after alignment masking (Table 2). Neighbor-Net graphs as well showed only marginal reduction of conflicts after alignment masking (Figure 3). Unmasked EST data did not yield well supported resolved trees. Most ALISCORE masked alignments led to clearly improved resolution of 'traditionally' recognized clades (e.g. Chelicerata, Hexapoda, Pancrustacea). If alignments were masked using GBLOCKS(all) or GBLOCKS(half), tree resolution increased likewise. Using the GBLOCKS(none) masking option did not improve resolution compared to other masked alignments (Figure 2). Considering bootstrap values as measurement of tree-likeness, GBLOCKS(all), GBLOCKS(half), and ALISCORE improved tree-likeness of the data (Table 2). Except for the default GBLOCKS(none) setting, Neighbor-Net graphs showed a substantial decrease of conflict after alignment masking (Figure 3).

##### Nucleotide data

Again, ALISCORE and GBLOCKS(all) masking improved tree-likeness of the 12S + 16S nucleotide alignments at the taxonomically ordinal level. ALISCORE outperformed GBLOCKS(all) and GBLOCKS(half) in all instances. GBLOCKS(none) clearly performed worst (Figure 2, Table 2).

## Discussion

**Figure 4:**
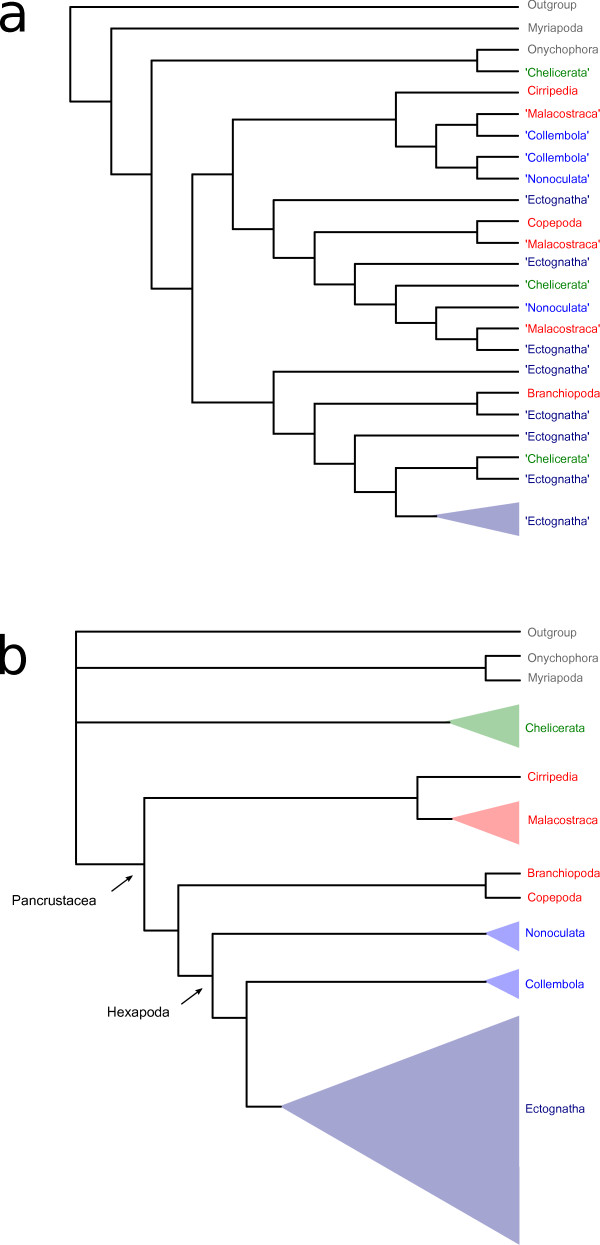
**Schematized cladograms inferred from the unmasked and masked EST data set**. Schematized cladograms (best ML trees, majority rule) inferred from the T-COFFEE aligned EST data set a) unmasked b) masked with ALISCORE considering selected clades. Quotation marks indicate non-monophyly of clades. Color code: Outgroup (tardigrades), onychophorans, myriapods: grey; chelicerates: green; crustaceans: red; hexapods: blue (proturans, diplurans, collembolans: royal-blue; ectognath hexapods: dark-blue).

Parametric and non-parametric masking methods were successful in identifying 'problematic' alignment blocks. In general, removal of these blocks prior to tree reconstruction improved resolution and bootstrap support. We interprete these results as an improvement in signal-to-noise ratio. For data set mtI and mtII, we assumed clade validity congruently to Talavera & Castresana [11]. For the EST data set, traditionally accepted clades were only recovered for masked data sets in contrast to the unmasked approach, e.g. Pancrustacea [19-27], Malacostraca [28-30], Hexapoda [19,20,22,25-27,31-35], Ectognatha [22,26,31,32,34,36] or Collembola [22,26,31-35], see Figure 4. A detailed review on these clades including morphological, neuro-anatomical and palaeontological evidence has been recently published in Edgecombe [37] and Grimaldi [38]. An improved data structure after alignment masking is also supported by more distinct split patterns (Figure 3).

Alignment masking further reduced sensitivity of tree reconstructions to different alignment methods. The method implemented in GBLOCKS has the potential to overestimate the extent of divergent or ambiguously aligned positions, especially in partial gene sequences and gappy multiple sequence alignments like EST data or rRNA loop regions. Masking with the GBLOCKS(none) option tended to result in suboptimal node resolution and support values (Figure 2 and Table 2, 3). In the case of the 12S + 16S rRNA data, GBLOCKS(none) masking even reduced signal strength. This phenomenon is clearly evident in Figure 3, where the split decomposition pattern appears most fuzzy in the GBLOCKS(none) Neighbor-Net graph. We conclude that the incongruence between GBLOCKS(none)- and remaining masked trees may have resulted from conservative and stringent default parameters settings of GBLOCKS, in which all gap including positions were removed and only large conserved blocks were left. While the higher amount of conflicting signal in unmasked multiple sequence alignments clearly based on noisy data, it seems that the GBLOCKS(none) masking discarded too many informative positions.

**Table 3:** Average percentage of resolved clades per data set

Data	Gb(none)	Gb(half)	Gb(all)	Al	Unm
mtI	91.7	91.7	93.8	93.8	83.8
mtII	58.3	56.7	58.3	60	58.3
EST	57.1	85.7	96.4	85.7	0
12S + 16S	16.6	86.1	88.9	97.2	77.7

Average percentage of resolved clades (selected) inferred from majority rule ML trees, averaged across different alignment methods. Values are given for all GBLOCKS profiles (Gb(none), Gb(half), Gb(all)), ALISCORE (Al), and Unmasked (Unm).

The ALISCORE and GBLOCKS(all) approach performed quite similar and best on all data sets. This demonstrates that even a predefined set of rules suffices to extract randomness within sequence alignments. Talavera & Castresana [11] showed this already in their extensive analyses of GBLOCKSs performance. The use of large data sets in phylogenomic analyses resulted in a tremendous increase of molecular data, but also in an increase of sampling error which could even bias seemingly robust phylogenetic inference [39]. Several such cases have been reported [40-42]. Therefore, it is important to establish a reliable alignment masking approach to cope with systematic errors in multiple sequence alignments. Our analyses showed that the sliding window approach will be a useful profiling tool to guide alignment masking.

ALISCORE optionally uses a BLOSUM62 or various PAM matrices to score differences between amino acid sequences, or a simple match/mismatch score for differences between nucleotide or amino acid sequences. It uses a simple modified Poisson model of character state change (called Proportional for amino acids [18], adapted for uneven base composition and sequence selection) in its resampling procedure to generate a null distribution of expected scores of randomly similar sequences. These scoring models and resampling processes are not very realistic, but however performed well in our analyses.

A recently published alternative approach, NOISY, uses a qnet-graph of sequence relationships to assess randomness of single positions [2]. The approach uses Monte Carlo resampling of single columns to compare fit of random data columns on a qnet-graph with the fit of observed data columns. The NOISY method appears as a fast and better alternative to the GBLOCKS approach, but a comparative analyses of its performance with the sliding window approach remains to be done.

## Conclusions

We demonstrate for empirical data that alignment masking is a powerful tool to improve signal-to-noise ratio in multiple sequence alignments prior to phylogenetic reconstruction. Masking multiple sequence alignments makes them additionally less sensitive towards different alignment algorithms. Our study also shows, that the scoring algorithm for amino acid data implemented in ALISCORE performs well.

The ALISCORE (parametric) approach is independent of *a priori *rating of sequence variation and seems to be more capable to handle automatically different substitution patterns and heterogeneous base composition.

It will be a matter of further analyses, whether an extension of the sliding window approach to more realistic likelihood models of change and Monte Carlo resampling will further improve the performance. However, it would be conceivable to implement a more explicit model based approach in GBLOCKS as well. The advantage of improved parameterizing GBLOCKS could be a significant gain in speed compared to the sliding window approach. The best approach should be the most efficient one in terms of computational time and increased reliability of trees, the latter one admittedly hard to assess.

## Methods

### Data sets

We used four different types of real data sets in combination with different alignment approaches, three mitochondrial (mt) and one nuclear (nu) data set (Fig. 1). Complete mt protein coding sequences of 11 genes were downloaded for eukaryotes from SwissProt and GenBank. Six genes (*COII*, *COIII*, *ND2*, *ND3*, *ND4L*, *ND6*) show high sequence variability compared to the less variable genes (*COI*, *Cytb*, *ND1*, *ND4*, *ND5*). The first mt data set (mtI) included protein sequences of all chosen mitochondrial genes of 17 taxa. The second mt data set (mtII) comprised the five less variable genes out of data set mtI but with 24 taxa, corresponding to Talavera & Castresana [11]. The third mitochondrial data set (12S + 16S) included nearly complete 12S + 16S rRNA sequences for 63 arthropod taxa. The nuclear data set (EST) was compiled from 51 mainly ribosomal protein coding genes from Expressed Sequence Tags (ESTs) of 26 arthropod taxa. These were selected from published (dbEST, NCBI) and unpublished EST data (Meusemann, v. Reumont, Burmester, Roeding, unpubl.). The data set comprised representatives of all major arthropod clades including water bears (Tardigrada) and velvet worms (Onychophora). A definitive tree of arthropods has not been established yet, therefore we restricted our comparison on tree resolution and bootstrap support values for selected clades. We remark that increased resolution and support might not reflect a real improvement of phylogenetic signal-to-noise ratio, but we consider this comparison as a good approximation in which the bootstrap values are used as approximation of tree-likeness in the data.

### Alignments

All genes were aligned separately, each data set using MAFFT 6.240 [14], MUSCLE 3.52 [15], CLUSTALX 1.81 [13], and T-COFFEE 5.56 [16] with default parameters. Since the number of taxa of the rRNA data was too high for T-COFFEE, PCMA 2.0 [17] was used instead which aligns more similar sequences with the CLUSTAL algorithm and less similar sequences with the T-COFFEE algorithm. Each alternative alignment was profiled once with ALISCORE and with all three possible gap predefinitions of GBLOCKS in which either no gaps (GBLOCKS(none)), all gaps (GBLOCKS(all)), or positions which have in less than 50% of sequences a gap (GBLOCKS(half)) are allowed. Thus, five different sets per alignment method were used in tree reconstructions: a) unmasked, b) three different GBLOCKS masked, and c) ALISCORE masked. This was conducted for all four data sets (mtI, mtII, 12S + 16S, EST). Using ALISCORE, alignments were screened separately with 2,000 randomly drawn pairwise comparisons and a window size *w *= 6. Within its scoring function gaps were treated like ambiguous characters on nucleotide level. On amino acid level we used the BLOSSUM62 substitution matrix. Positions identified by ALISCORE or suggested by GBLOCKS as randomly similar were removed and single genes were concatenated for each data set and each approach. Percentage of remaining positions after masking was plotted for each alignment and masking approach (Figure 1), in total for 1,104 single alignments (see additional file 1).

### Split Networks

Split decomposition patterns were analyzed with SplitsTree 4 [43], version 4.10. We used the Neighbor-Net algorithm [44] and uncorrected p-distances to generate Neighbor-Net graphs from concatenated alignments of each data set before and after exclusion of randomly similar sections.

### Tree reconstructions

Maximum likelihood (ML) trees were estimated with RAxML 7.0.0 [45] and the RAxML PTHREADS version [46]. We conducted rapid bootstrap analyses and search for the best ML tree with the GTRMIX model for rRNA data and the PROTMIX model with the BLOSUM62 substitution matrix for amino acid data with 100 bootstrap replicates each. Twenty topologies with bootstrap support values of all three GBLOCKS masked, ALISCORE masked, and unmasked alignments were compared for each single data set. Majority rule was applied for all GBLOCKS masked, ALISCORE masked, and unmasked topologies to investigate consistency of selected clades. Clades below 50% bootstrap support were considered as unresolved.

## Competing interests

The authors declare that they have no competing interests.

## Authors' contributions

PK and BM conceived the study, PK designed the setup and performed all analyses. PK and BM wrote the paper with comments and revisions from KM, BT, BMvR and JWW. Beta testing of ALISCORE and processing of ESTs was conducted by KM. Sequence data was provided by KM (ESTs, hexapods), JD (12S + 16S rRNA), and BMvR (ESTs, crustaceans). All authors read and approved the final manuscript.

## Supplementary Material

Additional file 1 **Table S1.** Detailed analyses results. Detailed results including lists of the percentage of remained positions after alignment masking per data set and alignment method. Given are all considered clades and corresponding bootstrap values (>50%) per data set, alignment method and (un)masked approach as well as all Neighbor-Net graphs. Click here for file
